# Mapping of affordability levels for photovoltaic-based electricity generation in the solar belt of sub-Saharan Africa, East Asia and South Asia

**DOI:** 10.1038/s41598-021-82638-x

**Published:** 2021-02-05

**Authors:** Sándor Szabó, Irene Pinedo Pascua, Daniel Puig, Magda Moner-Girona, Mario Negre, Thomas Huld, Yacob Mulugetta, Ioannis Kougias, László Szabó, Daniel Kammen

**Affiliations:** 1 European Commission, Joint Research Centre (JRC), Ispra, Italy; 2 European Institute of Innovation and Technology, Budapest, Hungary; 3 Technical University of Denmark, Kongens Lyngby, Denmark; 4 German Development Institute, Bonn, Germany; 5 University College London, London, UK; 6 Regional Centre for Energy Policy Research, Corvinus University of Budapest, Budapest, Hungary; 7 University of California at Berkeley, Berkeley, USA

**Keywords:** Energy access, Photovoltaics

## Abstract

Lack of access to modern forms of energy hampers efforts to reduce poverty. The provision of electricity to off-grid communities is therefore a long-standing developmental goal. Yet, many off-grid electrification projects neglect mid- and long-term operation and maintenance costs. When this is the case, electricity services are unlikely to be affordable to the communities that are the project’s primary target. Here we show that, compared with diesel-powered electricity generation systems, solar photovoltaic systems are more affordable to no less than 36% of the unelectrified populations in East Asia, South Asia, and sub-Saharan Africa. We do so by developing geo-referenced estimates of affordability at a high level of resolution (1 km^2^). The analysis illustrates the differences in affordability that may be found at the subnational level, which underscores that electrification investments should be informed by subnational data.

## Introduction

About 56% of the population of sub-Saharan Africa lacks access to modern forms of energy. The corresponding shares in East and South Asia are 3% and 11%, respectively^1,2^. For comparison, whereas the populations of Africa and China are similar in size, Africa’s installed electricity generation capacity is one-tenth of that in China^2^. The economic, social and environmental impacts of this shortage contribute to perpetuating poverty in these regions^3^. For this reason, providing electricity to unelectrified communities has been a long-standing policy priority of national governments, aid donor- and aid recipient-countries, and is an ambition incorporated in the Sustainable Development Goals set by the United Nations General Assembly. Under Sustainable Development Goal 7 (“ensure access to affordable, reliable, sustainable and modern energy for all”), an aspirational target has been set to ensure universal access to affordable, reliable and modern energy services by 2030^1^. Yet, progress in electrification has been slow, and has relied mostly on centralized generation and grid extension, a choice that is not always suitable for scattered rural communities^4^.

To provide electricity to unelectrified communities and meet the expected increase in demand of communities that already have access to electricity, at least 900 gigawatts of new electricity generation capacity will have to be installed over the next 30 years, a tenfold increase relative to current installed capacity across Africa^5,6^. However, lack of access to modern energy services is concentrated in rural areas, where 80% of the energy-poor live^7^. Therefore, investments will have to be provided through development aid^8^ and independent power producers^5^, who are mostly privately capitalized, because the credit ratings of national governments and public utilities in these regions are inadequate for raising the amounts of capital required to finance universal electrification. Such investments can only materialise if the following conditions are met^5,8,9^: credible power sector planning is introduced; financial risks are reduced, through the involvement of development-finance institutions and other risk-mitigation measures; and regulatory frameworks are reformed, to make them more conducive to attracting the required investments.

A large share of the communities that currently enjoy access to electricity can afford electricity tariffs (by one estimate, a share as large as 90%)^10^. However, affordability stagnates at the approximately 25% of the population that lacks access to electricity^10^. To make electricity affordable to all, potential short- and mid-term investment returns need to be specified for the full range of electrification options and, spatially, across individual communities.

In grid-connected communities in sub-Saharan Africa, insufficient generation capacity and inadequate transmission and distribution infrastructure result in frequent outages. These outages lead to increased use of diesel generators. The differences in energy-system behaviour have become even more evident in the disruptive lockdown associated with the COVID-19 pandemic. The lockdown caused carbon emissions to rise in African cities, in sharp contrast to the—temporary—drop in emissions in China, and the improvments in air quality in India’s largest population centres. Europe has also seen major reductions in emissions of carbon dioxide and nitrous oxide.

The increased use of diesel generators during the lockdown, to keep the economy afloat, brought about a shift in work patterns in many African countries, including Kenya and Nigeria, where many whitecollar employees have been asked to work from home. Although the informal sector represents a large part of the urban economy, and it has been greatly affected by the pandemic, the lack of statistics prevents an analysis of how the lockdown and its implications on electricity generation affected the informal sector. Also, the role of photovoltaic- and diesel- based electricity generation systems as complements to unreliable grids is not documented enough in the literature. In light of this, the analysis reported in this article focuses only in the unelectrified communities.

This study maps unelectrified communities where, measured in terms of current energy expenditure, off-grid electrification powered by renewable energy would be affordable. Solar photovoltaic (PV) off-grid electrification is advantageous from two points of view. First, it reduces maintenance and running costs relative to diesel powered electricity generation, thus making the technology affordable for a larger share of the population in the long term. Second, it reduces emissions of local air pollutants and contributes to mitigating the emissions of global-warming greenhouse gases. For 71 countries, the analysis identifies the unelectrified communities in which solar-powered electricity generation is a feasible option even when competing with low-priced diesel.

In the period between 2007 and 2017, off-grid electricity generation represented only a minor share of total installed capacity, not exceeding 10 megawatts in the countries for which data are available. The only exceptions were Bangladesh, Ethiopia, India, and Indonesia. This study argues that, on the grounds outlined above, there is a strong case for expanding solar energy-powered off-grid electrification.

The article contributes to the literature by putting forward an innovative methodology for assessing the affordability of electrification systems. Specifically, the article advances current knowledge in three ways:

- Inclusion of ability-to-pay and affordability considerations into the analysis of rural electrification technologies at a highly disaggregated (1 km^2^) resolution never provided before.
- Development of a robust, geo-referenced approach for identifying locations in which renewable energy-powered electrification systems represent a no-regrets option on all accounts. This approach draws in pioneering sub-national estimates of affordability levels, combined with population characteristics (population size and density).
- Provision of estimates for 71 countries, spanning all of sub-Saharan Africa and the South and East of Asia, which are home to 85% of the unelectrified population worldwide.
  Compared to previous work in this area (country^11^ or continent-wide level^12–14^) the article sets an analytical framework and tests it in a large number of countries. Not least, for the various parameters that affect the performance and sustainability of off-grid electricity generation systems, the analysis presented in the article highlights how plausible proxies can be obtained where data are lacking, thus inviting future work in this area. Finally, the article underscores that highly disaggregated geo-referenced estimates are indispensable to make policy and investment decisions in the area of rural electrification.

### Mapping unelectrified populations

Building on earlier work^4,15^, this study focuses on the 71 countries in Africa, East Asia, and South Asia that are home to the vast majority (85%) of unelectrified communities worldwide (“Methods”). It thus provides a nearly-comprehensive assessment of universal electrification.

To analyse and quantify the spatial distribution of the population lacking access to electricity, the study relies on 2014 and 2015 geo-referenced datasets in various formats and spatial resolutions. The following paragraphs describe the assumptions adopted to obtain disaggregated estimates of the size of the unelectrified populations in the countries under study.

**Figure 1:**
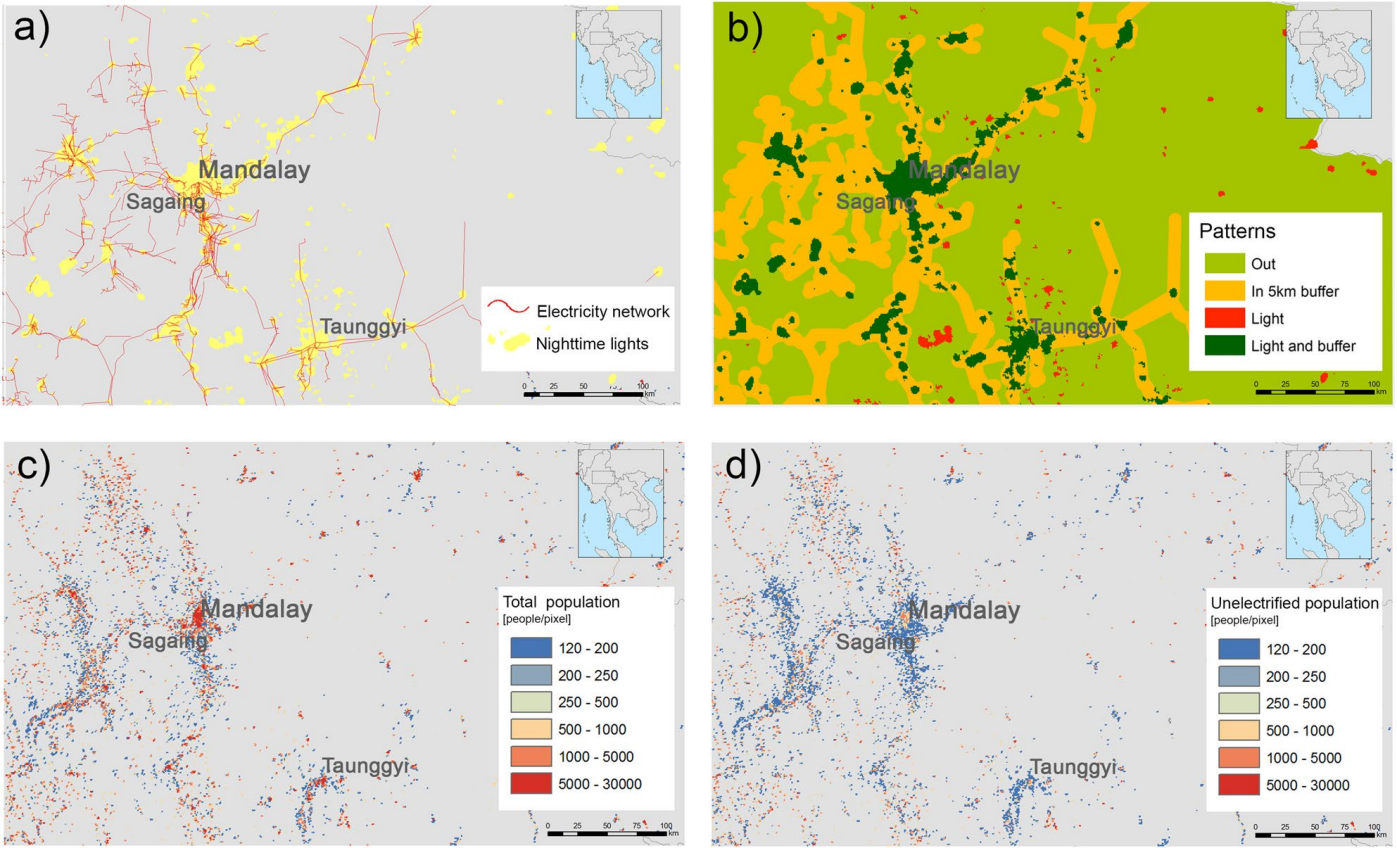
Data processing and spatial disaggregation of unelectrified populations: close-up on the Mandalay Region, Myanmar. (**a**) Identification of electricity network and nightlights data^17^. (**b**) Delimitation of zones according to proximity to the electricity grid and presence of nightlights. Dark green-coloured areas correspond to communities that are close to the existing electricity grid (5 km buffer), show nightlights, and have access to electricity. These communities correspond to electrified areas. Orange indicates communities that are within a maximum of 5 km of the existing electricity grid, but lack nightlights. These areas correspond to the last-mile energy-access population—that is, communities with no connection to the grid today, but in which grid connection is feasible. Red-coloured areas correspond to communities that show nightlights, but are far from the existing grid. Light green-coloured areas lack access to electricity (no nightlights and far from the grid). (**c**) Distribution of the total population (inhabitants per 1 km^2^ grid) based on the Global Human Settlement Layer (GHSL)^16^. (**d**) Distribution of the unelectrified population (inhabitants per 1 km^2^ grid) as estimated through the study’s calculations. Starting with the World Bank electrification rates^20^, the study uses the total population in each pixel and the zone—coefficients for each country group are included in Table 1—as proxies to spatially disaggregate the unelectrified population. The maps were generated using the following data, collected and processed by the authors: GHS POPULATION GRID—GHS-POP^16^ data, produced and made publicly available by the European Commission—JRC (https://ghsl.jrc.ec.europa.eu/data.php); Nighttime lights Version 4 DMSP-OLS^17^, produced and made publicly available by NOAA's National Geophysical Data Center (https://ngdc.noaa.gov/eog/dmsp/downloadV4composites.html); the Electrification access rates^20^ (EG.ELC.ACCS.ZS) made publicly available by the World Bank through The Open Data Portal (https://data.worldbank.org); and Electricity Grid Vector data publicly available by several sources^18,19^ ( OpenStreetMap, https://www.openstreetmap.org/ , NREL—Geospatial Toolkit https://www.nrel.gov/international/geospatial_toolkits.html and EnergyData https://energydata.info). The spatial classification was made and mapped using ArcGIS 10.6 (https://desktop.arcgis.com/). GIMP 2.10 (https://www.gimp.org) was used for image editing. **Sources:** World Bank^20^; Global Human Settlement Layer^16^; Authors' compilation based on the analysis.
- Second, electrification rates are estimated for each of the zones referred to above (Table 1). These estimates reflect socio-economic and technological differences across countries, consistent with related national-level data (Fig. 2).

To estimate the unelectrified population in each pixel (grid size: 1 km^2^) in each of the 71 countries, the analysis uses a gridded population dataset based on Global Human Settlements (GHSL) data^16^ and World Bank national-level estimates of electrification. The process involves two steps, as follows:

- First, a number of zones are defined (Fig. 1b) according to the absence or presence of nightlights^17^ and the existence of electricity networks^18,19^ (Fig. 1a). In some countries, this corresponds only to transmission lines (66 or 100 kV). In other countries, it corresponds to both transmission lines and distribution lines (11 kV).

**Table 1:** Criteria used to calculate the relative size of the unelectrified population (per pixel, depending on the type of zone where the pixel is located, and the corresponding country group).

Country group	Type of zone			
	In 5 km buffer (orange)	In light area (red)	Inside both (dark green)	Outside (light green)
	**Electrification rate (percent of population with access to electricity)**			
South East Asia	60	95	95	0
North Africa	90	80	100	90
Sub-Saharan Africa	10	60	95	0
South East Asia electrified	100	100	100	90

**Source:** Authors' compilation based on the analysis.

**Figure 2:**
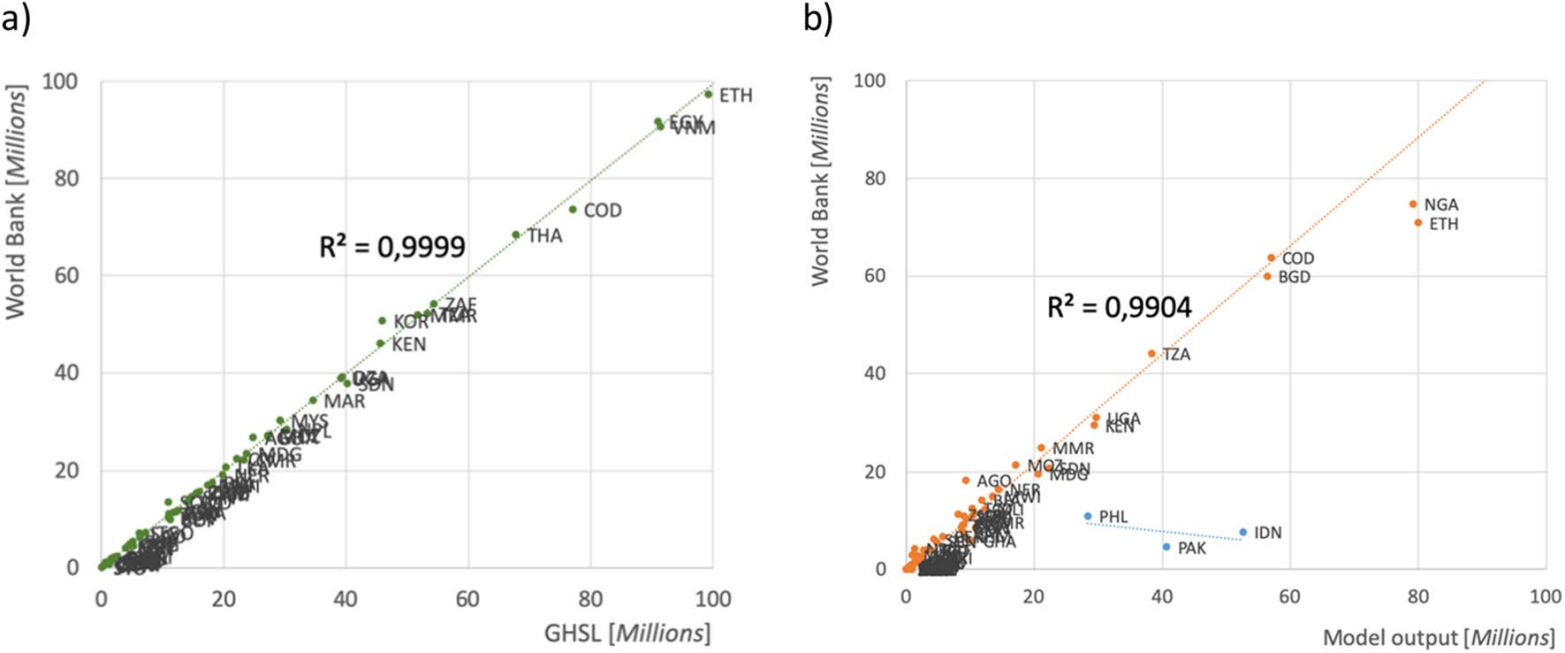
Statistical analysis of the spatial distribution data, by country. (**a**) Correlation between national-level data on total population^20^ and data on population per cell from GHSL^16^. (**b**) Correlation between data on the size of the unelectrified population at the national level^20^. Indonesia, Pakistan, and the Philippines are not considered in the adjustment because of large discrepancies in the reported data on the unelectrified population (“Methods”). Sources: World Bank^20^; Global Human Settlement Layer ^16^; Authors' compilation based on the analysis.

Table 1 illustrates the assumptions made concerning electrification rates, by country group and type of zone. The values are calculated through an iterative process that maximizes the correlation between (a) the most reliable data on people lacking access to electricity within each country and (b) the result of summing up the values of every cell across the entire country.

To illustrate the type and level of disaggregation of the estimates produced, Fig. 1 presents the results for a square area located in central Mandalay, Myanmar. Figure 1c shows the distribution of the population in the region, approximately 16 million people, of the total 51 million people in the country. Figure 1d shows the result of the spatial disaggregation of the unelectrified population in each pixel, using the electrification rate allocated at the relevant type of zone in the South East Asia row in Table 1.

## Electrification costs and avoided carbon dioxide emissions

Drawing on previous efforts to map the generation costs of off-grid technologies in Africa^4^, the study calculates site-specific electricity generation costs at a resolution of 1 km^2^. It does so for the two main electrification technologies: decentralized solar PV energy system minigrids and diesel-based minigrids.

The analysis also estimates annual investment costs, the share of the population that would use one technology or the other, and the carbon-dioxide emissions associated with the provision of universal access to all unserved communities. Such a calculation allows an assessment of both the maximum and the minimum rates of deployment of PV technologies under two scenarios: (a) high diesel prices and (b) low diesel prices.

### Techno-economic conditions for decentralized photovoltaic and diesel options

The analysis distinguishes two scenarios for the deployment of PV systems, corresponding to combinations of regulatory frameworks and market conditions that are (a) positive or (b) negative for solar PV installations. Favourable PV conditions are consistent with the first pathway in the so-called shared socioeconomic pathways (SSP1). SSP1 refers to the first pathway in a set of scenarios labelled shared socioeconomic pathways. These scenarios provide narratives of plausible future changes in socioeconomic parameters^21^. They are used to foster comparability among prospective studies on climate change management. SSP1 describes a future that shifts strongly towards low-emission technologies and the sustainable management of natural resources. In the favourable PV conditions scenario, diesel prices in each country are fixed at the highest retail prices in the last decade (2012 prices), including national taxes and subsidies (“Methods”). The parameters used to estimate the production costs associated with solar PV minigrids reflect current component prices (including storage), replacements, and Operation and Maintenance (O&M) for a lifetime of 20 years. Table 2 lists the component prices of recently installed PV minigrids^22^ and the settings for the PV battery optimization process^23^. Following recommendations for this type of energy-infrastructure cost–benefit analysis^24,25^, the discount rate was set to 5% (“Methods”).

**Table 2:** Parameters used for the calculation of production costs of PV-based decentralized systems.

Parameter	Value	Unit
PV module price	0.95	US$/W_p_
Balance of system price	1.15	US$/W_p_
Li-on battery price	400	US$/kWh
System lifetime	20	years
Battery lifetime	10	years
Discount rate	5 percent	
Consumption during daytime	2/3	
Days with power loss	5 percent	

**Sources:** Moner-Girona et al.^22^; Huld et al.^23^.

Adverse PV conditions are consistent with SSP3. Under SSP3, regionalization wins out over integration; current high-fertility countries experience high population growth, and oil-price volatility becomes the norm. This scenario assumes that investment decisions favour low-cost diesel. For this reason, the diesel price of February 1, 2016 is used—that is, the price of diesel immediately after the emergence of one of the lowest values of Brent in the previous ten years^26,27^ (see “Methods”). Under these conditions, decisions about electrification are likely to be dominated by three factors. First, PV minigrids exhibit higher up-front costs compared with diesel-based minigrids. Second, high population-growth rates result in stronger demand for quick and cheap electrification, relative to the favourable PV conditions. In this reasoning, higher interest rates lead to lower, more widely discounted future operational costs that are associated with the purchase of fuel. Third, the growth in demand for diesel leads to increased diesel prices in the mid term. The national retail diesel prices of 2012 are used^28^.

### Least-cost electrification options

For each of the two technologies, estimates are produced of the costs and annual greenhouse-gas emissions associated with providing access to electricity to the entire unelectrified population in each square kilometer cell (“Methods”). An annual residential consumption of 1250 kWh per household is assumed^1^ (“Methods”). For each cell, technology-specific costs are compared, and the least-cost option is selected. Figure 1 offers a close-up on a region of south-east Asia. It compares the results on the least cost option in the case of (a) favourable and (b) adverse PV conditions. These estimates highlight how volatile oil prices affect the relative competitiveness of the two technologies and ultimately lead to a lock-in on diesel-powered systems if diesel prices are low. In this situation, the areas in which PV is comparatively more competitive are smaller, relative to the results under the favourable scenario, wherein the deployment of PV-fuelled systems reaches a peak.

**Figure 3:**
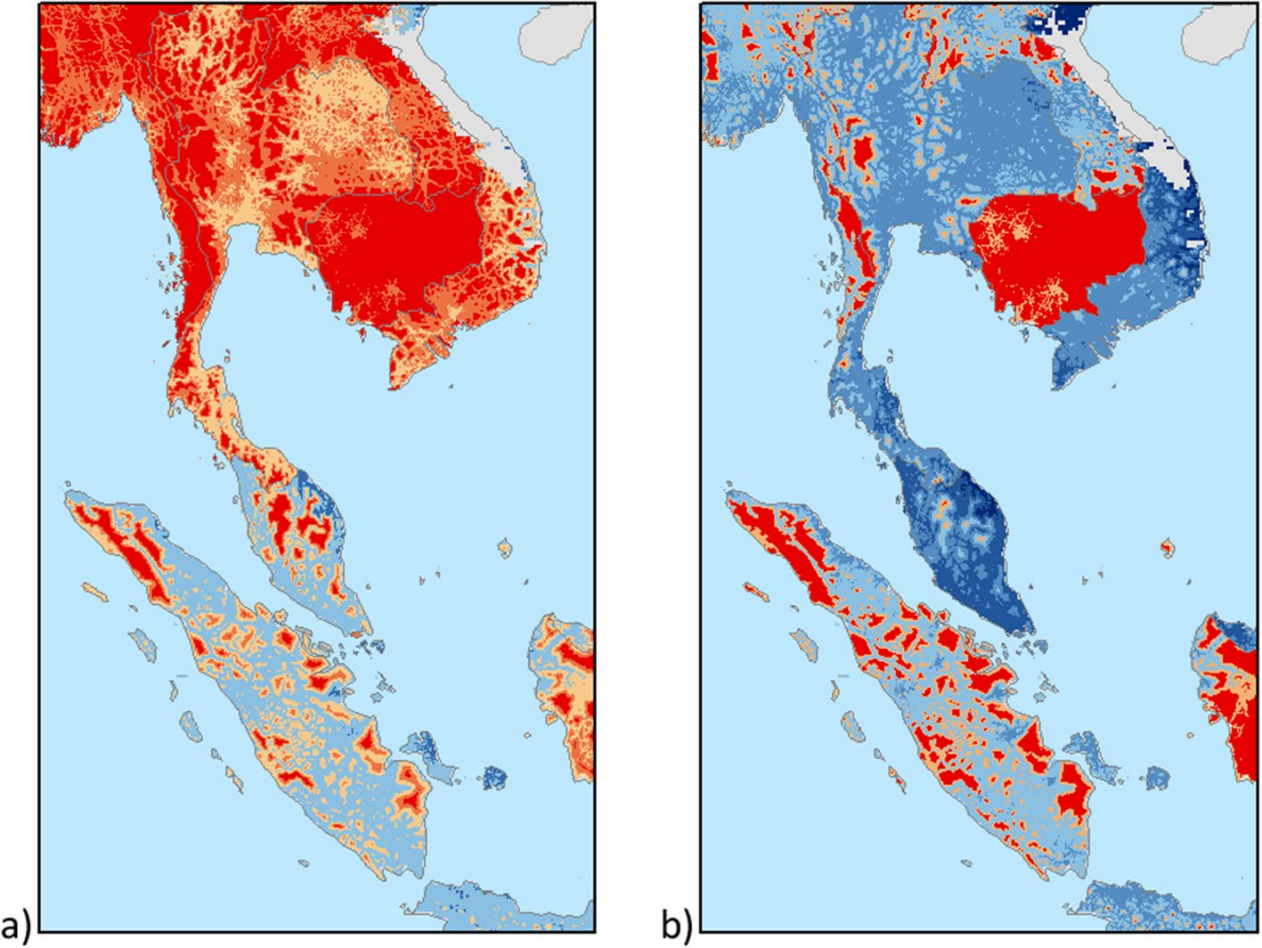
Competitiveness of PV-based (red) and diesel based (blue) minigrids in the Indochinese peninsula under (**a**) favourable and (**b**) adverse scenarios for PV. Red-coloured areas are those in which PV minigrids are cheaper. Blue-coloured areas are those in which diesel-based minigrids are cheaper. The higher the contrast, the larger the difference in cost estimates. ArcGIS 10.6 (https://desktop.arcgis.com/) was used to calculate the values and generate the map. GIMP 2.10 (https://www.gimp.org) was used for image editing.

### Environmental performance of photovoltaic and diesel-based systems

With regard to health and environmental impacts, PV-based electrification systems unambiguously outperform their diesel based counterparts. Indeed, in the adverse PV scenario, carbon-dioxide emissions are greater by a factor of 6, and local air quality is comparatively much lower^29^.

The study results show the two main impacts of diesel price volatility (Table 3). First, the share of the population that relies on PV declines from 78 to 36%. Second, annual O&M costs rise from 21 to 77%. Both impacts reflect the consequences of investing in diesel-powered systems, a decision that locks in the technology and the associated impacts over the mid term. Shifting away from diesel would require major efforts in communication with stakeholders, because diesel is well known and widely used in the countries concerned and, for this reason, benefits from such incumbency position.

**Table 3:** Population relying on PV, the associated carbon dioxide emissions, and associated annual costs.

	Favourable conditions for PV	Adverse conditions for PV
Population without access that would rely on PV (% / million people)	78 / 1089	36 / 443
Associated carbon dioxide emissions annually (MtCO_2_)	58	305
Associated annual costs (US$ billion)	69.35	91.51
Associated O& M (% total annual costs / US$ billion)	21.5 /14.93	77.4 / 70.78

**Source:** Authors' compilation based on the analysis.

### Identifying areas for no-regret investment in photovoltaic electricity generation

**Figure 4:**
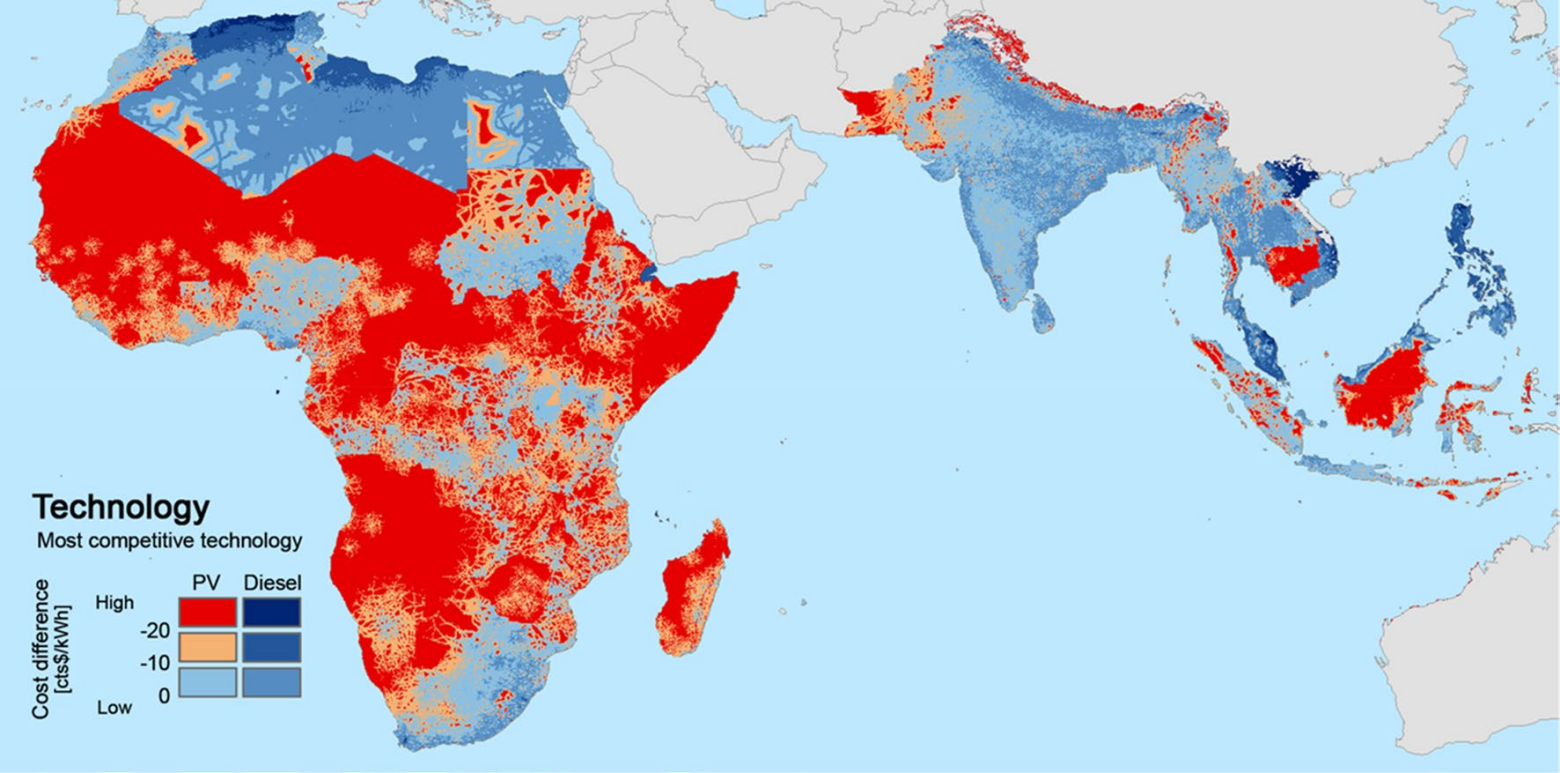
Location-specific estimates of the relative cost of solar PV versus diesel-based minigrids in the adverse PV conditions scenario. Red-coloured areas are those where PV minigrid investments represent no-regrets options. In these areas, even under adverse PV conditions, PV generation costs are at least US$0.20 /kWh cheaper than diesel generation. ArcGIS 10.6 (https://desktop.arcgis.com/) was used to calculate the values and generate the map. GIMP 2.10 (https://www.gimp.org) was used for image editing.

The geo-referenced estimates introduced in the previous sections allow the identification of areas in which PV investments would represent a no-regret option, not accounting for the planned extension of electricity grids. No-regret option refers to areas where, even under adverse PV conditions, the cost of electricity generation using PV is more than US$0.20 per kWh cheaper than the corresponding cost of using a diesel generator. Figure 4 illustrates the results under the adverse PV conditions scenario. In the red areas, where 443 million people live (35% of the population considered in the study), PV minigrids represent the least-cost electrification option.

The analysis covers an unelectrified population size of roughly 1.2 billion people. It finds that PV minigrids is the no-regret option for the provision of electricity to 177 million people, and that this option is nearly competitive (a difference of less than US$0.10) for another 266 million people—that is, slightly more than one-fifth of the population in the study.

Most energy-poor communities (92% of the 1.2 billion people) are concentrated in only 27 of the 71 countries under study. Table 4 provides estimates for these 27 countries (Table S.4 in the Supplementary Information shows the data for all countries). These estimates show that, as a no-regret option, PV ranges from negligible (mostly in Asia) to dominant. The countries with higher percent of unelectrified population living in areas where PV is a no-regret option (third column) are mostly those with high diesel prices (in 2012 or in 2016 or in both, see Sect. “Techno-economic conditions for decentralized photovoltaic and diesel options”) e.g. Burkina Faso, Angola and Malawi. In countries with big fuel subsidies, the situation is reversed and low percent of unelectrified population lives in no-regret PV areas. These low level fuel taxes are hard to explain on social or environmental grounds. Economic policy instruments to level these rates would create revenues for governments and would increase the competitiveness of PV in these countries.

**Table 4:** Decentralized PV as a no-regrets investment option, by country.

Country	Unelectrified population	Population in areas in which PV is a no-regret option	
	(million)	(million)	(% of unelectrified population)
Philippines	31.7	0.000	0.000
Bangladesh	71.5	0.003	0.004
India	294.8	1.1	0.4
Myanmar	26.2	0.1	0.5
Nigeria	93.2	1.0	1.1
Sudan	26.2	0.4	1.7
Pakistan	54.8	2.0	3.6
Democratic Republic of Congo	66.2	2.8	4.3
Uganda	34.7	1.9	5.6
Indonesia	54.1	4.1	7.6
Mozambique	20.4	2.0	9.6
Cameroon	13.1	1.3	9.7
Tanzania	44.7	4.4	9.9
Kenya	34.0	3.9	11.4
Niger	17.2	4.4	25.5
Zambia	9.5	2.7	28.1
Madagascar	25.0	7.1	28.5
Ethiopia	94.0	27.9	29.6
Burundi	10.6	3.9	36.9
South Sudan	12.2	5.6	45.9
Chad	12.5	6.0	47.6
Malawi	16.1	9.2	56.7
Zimbabwe	11.8	7.0	59.4
Mali	14.8	8.9	60.6
Burkina Faso	13.8	9.6	70.0
Angola	10.9	8.5	78.2

**Source:** Authors' compilation based on the analysis.

## “Low-hanging fruit” PV areas and the importance of affordability

The estimates presented thus far correspond to the share and spatial location of unelectrified communities in which PV minigrids represent a no-regret option purely from the economic perspective. However, comparatively lower costs alone will not necessarily lead to successful electrification interventions. Affordability must also be taken into account.

Electrification programmes incur two types of costs: up-front costs and O&M costs. After an initial period, the latter are borne by the programme beneficiaries. It follows that, if the programme beneficiaries cannot afford these costs, programme objectives will not be achieved. Because universal access to electricity targets the poorest areas in the world, it is imperative that O&M costs be low.

In light of the above, the analysis breaks down cost estimates into two components: up-front costs and O&M costs. It is assumed that the former will be borne by a public entity, such as a national government, an aid agency, or a development bank, and that the latter will be borne by beneficiaries of the electrification programme. It is worth noting that, most often, financial appraisals focus on up-front costs, because discount rates minimize the weight of O&M costs^30^. They do so even if ignoring O&M costs likely results in a system that is underutilized at best. A large number of projects financed by independent power producers are in this situation^31^.

Drawing on field data and conservative assumptions about the operating costs of a PV system, it is estimated that the O&M costs associated with PV minigrids represent one-tenth of the total costs of the system^22^. For diesel-based minigrids, the estimate is that US$0.01 per kWh covers the initial capital cost, accounting for the commercial price and the average lifetime of the 4–15 kw diesel generators. O&M costs account for the rest. This cost component accounts for the diesel consumption associated with the production phase, including the price increase associated with transportation from the distribution hub to the consumption location. In the case of diesel-powered systems, O&M costs are much larger, relative to the cost of PV-based systems.

To assess affordability, the study estimates two parameters: the running costs of electricity and the level of electricity expenditure that households can afford. Estimates of the latter are calculated under the assumption that, provided that the same services are obtained and the overall available expenditure is not exceeded, the choice of fuel is inconsequential to households. This assumption ignores the substitution between energy and other goods purchased by households.

**Figure 5:**
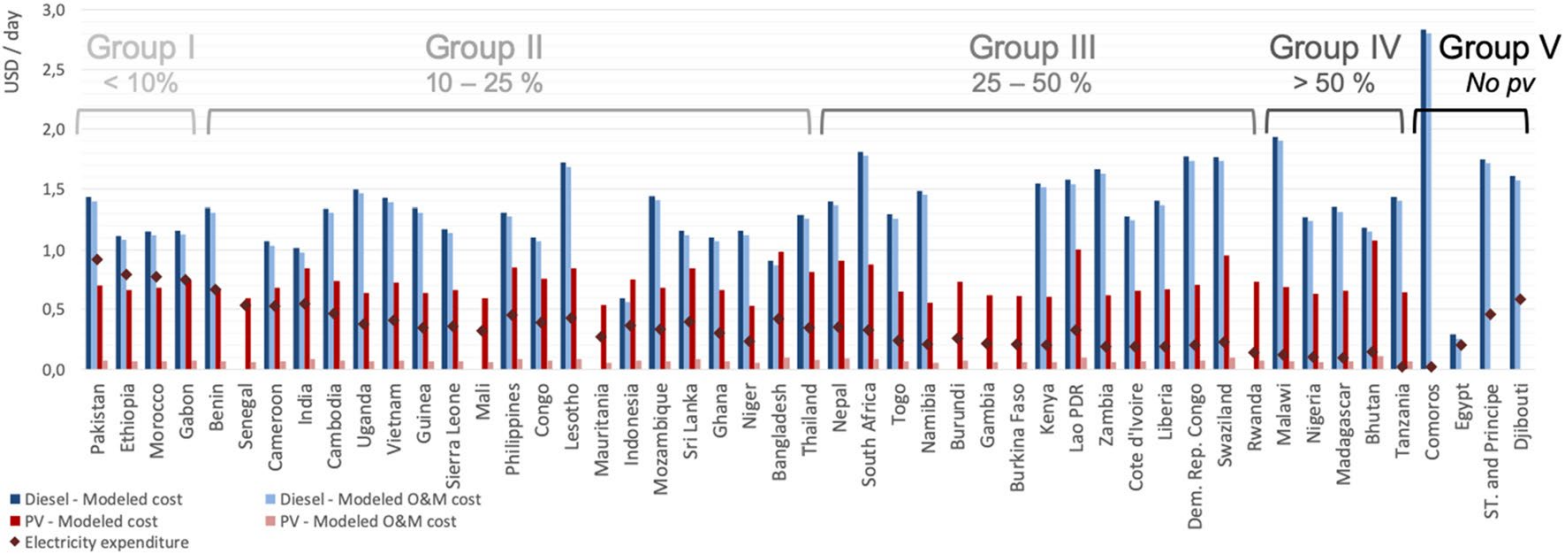
Modelled electricity generation costs—total and O&M—versus potential daily expenditure on electricity per poor household per day. These cost estimates are calculated under the adverse PV conditions scenario and clustered in five groups, according to the ratio between the potential daily expenditure on electricity and the modelled generation cost (total and O&M). Blue columns show average diesel-based electricity costs per country (O&M costs are presented in light blue), and red columns show average PV electricity generation costs (O&M costs are presented in light red). Rhombus sow the average potential daily expenditure on electricity per country. Group I clusters the countries where this expenditure is greater than the generation cost per day. Stated differently, in these countries electricity is more affordable among poor households. At the other extreme, in Group IV, electricity generation costs per day are at least 50% higher than the potential daily expenditure on electricity. In other words, in these countries poor populations face more difficulty in covering the cost of electricity. If the competing technology is cheaper across all locations within the country, the modelled cost (either PV or diesel) is not shown. **Source:** Authors' compilation based on the analysis.

Figure 5 shows two types of estimates: the cost of electricity, including the share that corresponds to O&M costs, and the potential daily expenditure on electricity of a poor household^32^. The latter is drawn from the World Bank’s Global Consumption Database^32^, a freely accessible database on household consumption patterns in developing countries (for a fuller description, see “Methods”). The gap between the two values reflects the extent to which the poorest percentile of the population in a given area can afford electricity (“Methods”). Figure 5 distinguishes between diesel- and PV-powered electricity generation systems.

Figure 5 illustrates two issues. First, the relevance of considering affordability in contextualizing the options for achieving universal electricity access in poor areas. Indeed, the results of the analysis reveal five clusters of countries in terms of the ratio of potential daily expenditure on electricity to cost, thus reflecting the difference in the expected ability to pay. The closer the modelled costs are to the actual energy expenditures in the rest of the country, the higher the market up-take that can be expected. Second, the differences in O&M costs per household, and in technology between countries. In almost all countries, poor households can afford the daily O&M cost of PV systems, but not the O&M cost of their diesel counterparts.

The study uses the phrase “low-hanging fruit” to describe communities that (a) are in areas in which PV constitutes a no-regret investment option, (b) are wealthier than the average in the country, and (c) include unelectrified populations, that is, potential new customers, representing a larger share of the total than the average in the country. Identifying such communities is useful because it provides information on the socioeconomic profiles of potential customers, an information that can be used to inform the design of an electrification project, with a view to raising its effectiveness.

**Figure 6:**
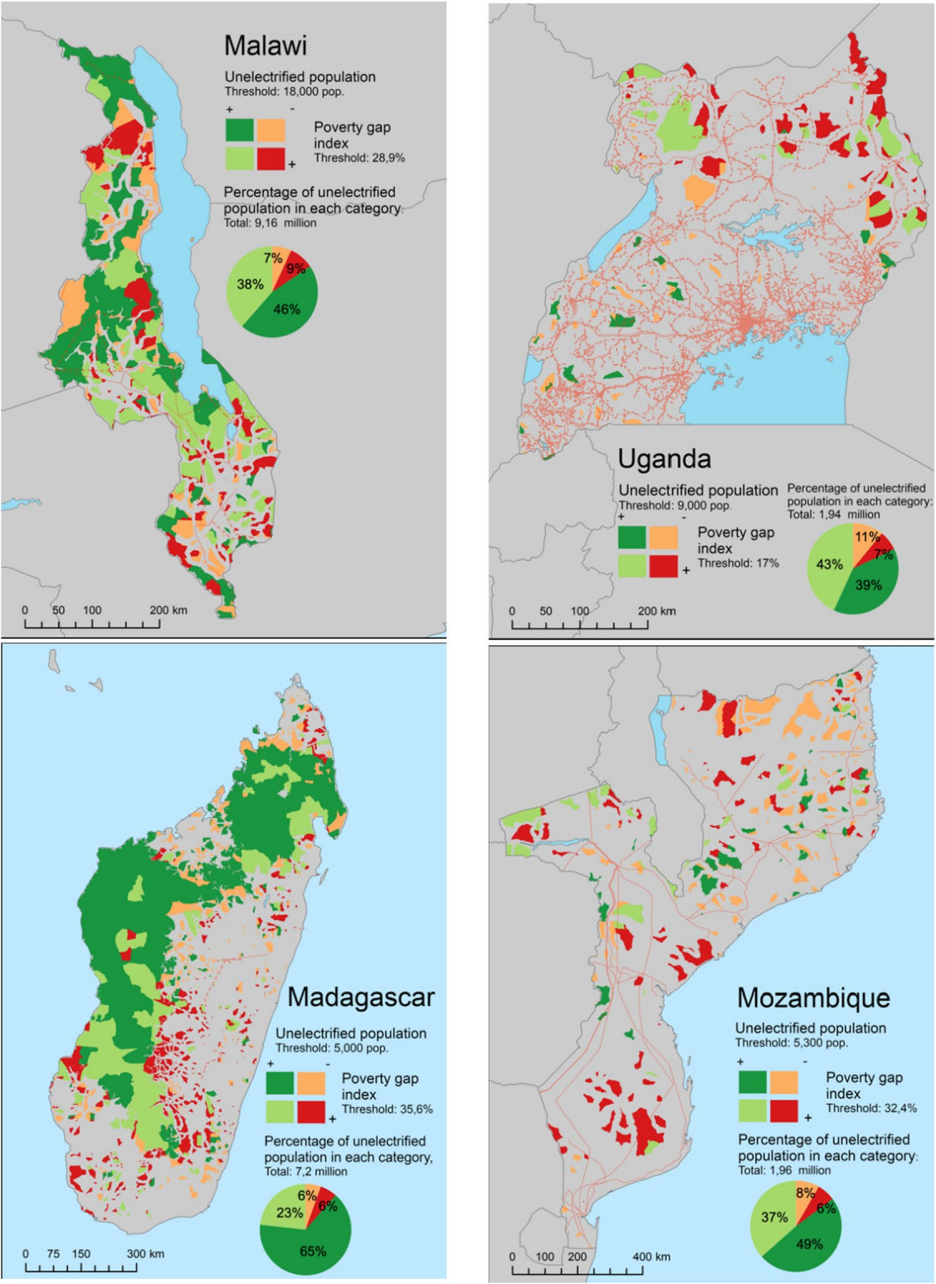
“Low-hanging fruit” PV areas in (**a**) Malawi, (**b**) Uganda, (**c**) Madagascar and (**d**) Mozambique. The analysis is restricted to no-regret PV option areas. Dark green-coloured areas correspond to “low-hanging fruit” areas, that is, communities with larger-than-average unelectrified populations (potential new costumers) that are wealthier than the average in the country. Dark red-coloured areas corresponds to those areas with smaller unserved populations that are poorer than the average. The values of specific thresholds are detailed in the maps. The maps were generated using the following data, collected and processed by the authors: the dataset with the disaggregated unelectrified population (described previously in “2. Mapping unelectrified populations”) , Administrative Level 3 Units, from GADM (https://gadm.org/) and the Poverty Gap Index—FGT1 at sub-national level from from CIESIN—Columbia University (for Mozambique, Malawi and Madagascar, https://sedac.ciesin.columbia.edu/data/set/povmap-small-area-estimates-poverty-inequality) and from World Resources Institute (Uganda, http://datasets.wri.org/dataset/uganda-rural-poverty-data-2005). Software used: ArcGIS 10.6 (https://desktop.arcgis.com/) and GIMP 2.10 (https://www.gimp.org) for image editing.

To identify and rank PV areas that represent low-hanging fruit areas, and lacking disaggregated affordability data, the study relies on subnational data drawn from the so-called poverty gap index. The poverty gap index can be defined as the average shortfall between actual household welfare status and the poverty line, expressed as a share of the poverty line^33^. Figure 6 shows the results for Madagascar, Malawi, Mozambique and Uganda, the few countries where these data are openly available. Examining only the areas in which PV constitutes a no-regret option, the analysis assigns to each area one of four categories defined by combining the poverty gap index and the size of the unserved population above or below the calculated country threshold.

Figure 6 reinforces the importance of affordability, which is also illustrated in Fig. 5. Simply stated, decisions concerning investments in electrification programmes cannot rely exclusively on average expenditure data at the national level. Indeed, such decisions should also be informed by subnational-level affordability data.

The high-resolution data presented in Fig. 6 for four countries provide a fuller and more reliable context-specific picture of customer affordability in these countries: while national-level data indicate that the O&M costs of PV may be affordable for 60% of the people living in the areas described as low-hanging-fruit, subnational data are needed to judge whether or not the remaining 40% of the populations in these areas can afford the O&M costs of PV systems. In such low-income communities, complementary pro-poor financing schemes would be required.

## Conclusions

For 71 countries and at a high resolution, the article reports a methodology that can be used to identify communities that can afford PV minigrids. These communities are a no-regret option for PV investors, because PV is the cheapest option, even under unfavourable PV conditions. Together, these areas are home to a large number of people who can afford the electricity tariffs associated with PV minigrids. Investing in PV technologies rather than diesel-based technologies would therefore be justified on two accounts: (a) the O&M costs associated with the electricity generation technology, and (b) health and environmental concerns.

At present, most national investment decisions on electrification are shortsighted. They often put too much weight on up-front costs, even if the magnitude of these costs may eventually render the investment futile. The findings reported here are directly relevant to governments in low-income countries as well as to development agencies and banks. Specifically, the methodology used, which is extensively documented in the methods section, could be used to inform the design of future electrification programmes, especially those targeting rural areas with low purchasing power.

Investing in PV minigrids in communities where the technology represents a no-regrets option could serve two purposes. First, these communities would be provided with affordable and sustainable electricity. Second, such an investment could help turn the tide on the investment-decision paradigms sketched above. Ultimately, the findings reported in this article suggest that investments in electrification led both by the public sector and by independent power producers will generate suboptimal results if they are framed around decision criteria that neglect O&M costs as well as health and environmental concerns. As more granular consumption data become available, this methodology can be used to study the issue of affordability at higher spatial resolutions.

## Methods

### Geographical coverage

The geographic coverage of the study is the area of the 71 countries depicted in Fig. 4 in the main text. The roles of these countries in achieving Sustainable Development Goal 7 vary substantially. Official databases on access to electricity globally show important discrepancies. The difference between 2014 estimates of electricity acces by the International Energy Agency (IEA) and the World Bank is approximately 200 million people worldwide^1^.The IEA’s higher estimates are based on utility connections, while the World Bank’s estimates are based on household surveys.

The Global Tracking Framework, an intergovernmental effort aimed at tracking progress with three energy sector-related goals, including access to electricity, identifies 20 “high impact countries” in light of their current low rates of electricity access and large populations. To this list, the study added another 7 countries for two reasons. First, Burundi, Cameroon, the Philippines, Zambia, and Zimbabwe were added because the unelectrified population in these countries is estimated at over 9 million people. Second, Indonesia and Pakistan were added because of the large discrepancy in the estimates of unelectrified population by the IEA and the World Bank. Concerning Pakistan, the World Bank reports an electricity access rate of 97.5%, while the IEA reports 72.5%. Regarding Indonesia, the respective estimates are 97.0% and 84.0%. The IEA reports that nearly 50 million people in Pakistan lack access to electricity, whereas the World Bank puts that figure at 4.5 million. In the case of Indonesia, the respective reported estimates are 40 million and 7.6 million people.

The 27 countries (20 identified as “high impact” by the Global Traking Framework, and the 7 we added) were given priority during decisive tasks and outcome validation.

Listed in decreasing order by the size of the unelectrified portion of the population, the 71 economies included in the study are India, Ethiopia, Nigeria, Bangladesh, the Democratic Republic of Congo, Tanzania, Kenya, Uganda, Myanmar, Mozambique, Sudan, Angola, Niger, Madagascar, Malawi, Burkina Faso, Chad, the Republic of Korea, the Philippines, Zimbabwe, Mali, South Sudan, Pakistan, Indonesia, Zambia, Burundi, Cameroon, Benin, Bhutan, Botswana, Central African Republic, Côte d'Ivoire, the Republic of Congo, the Comoros, Cabo Verde, Djibouti, Algeria, the Arab Republic of Egypt, Eritrea, Western Sahara, Gabon, Ghana, Guinea, Gambia, Guinea-Bissau, Equatorial Guinea, Cambodia, the Lao People’s Democratic Republic, Liberia, Libya, Sri Lanka, Lesotho, Morocco, Mauritania, Mauritius, Malaysia, Namibia, Nepal, Rwanda, Senegal, Singapore, Sierra Leone, Somalia, São Tomé and Príncipe, Swaziland, the Seychelles, Togo, Thailand, Tunisia, Vietnam, and South Africa.

### Diesel prices

This study distinguishes two types of deployment conditions for photovoltaic (PV) systems. First, the favourable PV conditions that are consistent with the first pathway among the shared socioeconomic pathways, labelled SSP1. In this pathway, the future shifts strongly towards environmental protection and sustainable resource use. For this scenario, national retail diesel prices of 2012 are considered representative of the first half of the decade^28^. During this period, diesel prices were stable at a high level and reached the highest value of 2010–2020 in February 2012. National-level taxes and subsidies are included in national diesel prices (Table S.2).

The adverse conditions for PV systems are consistent with SSP3 or the regional rivalry–fragmentation SSP. In this case, regionalization wins out over globalization; current high-fertility countries experience high population-growth rates, and oil-price volatility becomes the norm. To replicate this second set of conditions, it is assumed that investment decisions are based on low-cost diesel. The lowest oil prices in 2010–2020 were recorded in the second quarter of 2020 because of the drop in demand caused by the lockdowns associated with the COVID-19 emergency and the price war between the Russian Federation and Saudi Arabia. Oil prices showed remarkable volatility, ranging between US$20 and US$40 per barrel (an average of US$30 per barrel). The low prices were only recorded in January and February 2016, when the price of Brent fell to around US$29 per barrel. Because retail fuel prices in the second quarter of 2020 were not available at the time of the analysis, country values of February 2016^26^ have been selected as input to recreate the adverse scenario.

If accelerated policy ambitions drive electrification-technology decisions in the near future, the prevailing financial project appraisal will make the high up-front investment option (the PV minigrid) more expensive because the higher interest rate will discount the future fuel cost. That decision, together with high population growth rates, makes diesel the most competitive alternative for more and more communities. However, higher demand would lead to an increase in diesel prices, and all initial investments would then have been locked in on unsustainable resources. For the rising diesel prices, the national retail diesel prices of 2012 have been used^28^.

### Cost calculation

Location-specific estimates of the cost of electricity from PV minigrids and from diesel generators were calculated following the methodology described in^4^.

To calculate the levelized cost of electricity (US$ per kWh) for PV minigrids, it was assumed that two-thirds of the electricity are consumed during daytime (for the load profile, consult^23^). The algorithm used in the calculations takes into account the daily PVGIS solar irradiation data, an optimized value of the PV array size and the battery size, and the calculation of the system performance ratio. The assumptions used for the analysis are as follows.

- The size of the system is minimized for a given electricity consumption, to guarantee a certain availability of power; i.e. in this analysis, the system was designed not to run out of energy on more than 5% of days.
- The daily energy consumption pattern is such that two-thirds of the energy is consumed during daytime and one-third during the evening and during nighttime.
- The PV array size is calculated for a nominal desired daily consumption, with both PV array size and battery size varying geographically, so as to satisfy this consumption.
- The performance ratio is assumed to be 70%, a little lower than typical grid-connected systems, due to the additional losses in the batteries.
- The battery discharge depth is 70%, which assumes specialized solar system batteries (AGM).The battery discharge depth is 70%, which assumes specialized solar system batteries (AGM).
- PV lifetime is twenty years and battery lifetime is five years.
- The price of the PV modules is taken as US$ 950 per kWp and US$ 1150 per kWp for installation and balance-of-system (BOS) components. Battery prices are estimated as US$ 400 per kWh for Li-on batteries. Operation and maintenance costs are assumed to be 2.5% of the price of the PV and BOS for each year of operation.
- Cash flow: 5% discount factor.

Following the recommendations of various international institutions^24,25^, the value of 5 per cent has been used as discount factor. When conducting cost–benefit analysis of energy infrastructure where benefits are clearly public, using social discount rates (in the range of 3 to 5%) is recommended^24,25^, even when the investments are private. The additional reason for applying a social discount rate is that it can be expected that the initial schemes will be guaranteed by international organizations (such as bilateral and multinational donors and domestic governmental schemes) to secure affordability for the end-users. These incentives cover a large part of the financial risks in the initial (trial) period.

With regard to diesel based electricity, fuel consumption is responsible for the largest share of the total cost. To estimate location-specific operating costs for diesel generation, the national diesel price was combined with the transport cost of diesel (derived from the travel time data contained in the Accessibility Map^34,35^) and calculated in three steps:

**a) Transport cost** (US$ per litre). For our model, we assume^4^ that the diesel is transported form the nearest major town to the consumption point. The values we use reflect the vehicle fleet in the study area. The routes are usually covered by minibuses and converted pickups that are imported and reconditioned and typically five years old at least. Several factors affect the supply and affordability of rural transport services in Africa^36^. Key among them, according to Dennis et al.^36^, is the fact that conventional vehicles designated to operate at high speeds on paved roads operate ineficiently at low speeds on rural earth roads, resulting in high fuel consumption and high emissions. The added cost due to transport is estimated using the following equation:

1 $${\text{P}}_{{\text{t}}} = {\text{ 2P}}_{{\text{d}}} \times {\text{ct}}/{\text{V}}$$

where *P*_*d*_ is the national retail cost of diesel; *c* (litre per hour, set to 14) is the transporting vehicle’s diesel consumption per hour; *t* (hour) is the transport time, and *V* (litre, set to 300 l) is the volume of diesel transported. A factor of 2 is added, to account for the drive back (since we assume dedicated transport).

**b) Production cost** (US$ per kWh) is calculated as:

2 $${\text{P}}_{{\text{p}}} = \, ({\text{P}}_{{\text{d}}} + {\text{P}}_{{\text{t}}} )\times{\eta}$$

where *P*_*d*_ is the national retail cost of diesel; *η* is the conversion efficiency of the generator (set to a value of 0.286 l per kWh).

**c) Cost of electricity** (US$ per kWh). To the production cost, we add the cost of labour, maintenance and amortization, estimated in US$ 1 cent per kWh (used in commercial prices and average lifetime of 4 to 15 kW diesel generators).

### Emissions calculation

The study produces estimates of the emissions of electricity produced by diesel generators. Account is made for both electricity production^4^ and fuel transport.

**a) Production emissions:** The emissions released by diesel generators as they burn diesel fuel to produce electricity are modelled as follows:

3 $${\text{E}}_{{\text{p}}} = {\text{ Dy}}_{{{\text{hh}}}} \times {\text{E}}_{{{\text{fd}}}} \times {\text{HH}}_{{{\text{pix}}}}$$

where *E*_*p*_ represents the emissions released during electricity generation; *Dy*_*hh*_ is the yearly demand per household (1,250 kWh a year, or the threshold between Tier 3 and Tier 4, was used); *E*_*fd*_ is the emissions factor for a diesel generator (the value of 1.27 kg of carbon dioxide per kWh from Jakhrani was used^37^); and *HH*_*pix*_ is the number of households per pixel in the raster. For now, the consequences are not being taken into account of varying the rated power of the required diesel generator per location, which should be as close as possible to the load demand, even though efficiency is inversely proportional to its rated power, the fuel consumption rate and carbon dioxide emissions^37^.

**b) Transport emissions:** To maintain coherence with the model used to calculate costs^4^, the hypothesis adopted is that the diesel is transported from the nearest major town to the location of the demand. Accordingly, values are used that may more closely describe the vehicle fleet in the study area. The routes are usually covered by minibuses and converted pickups that are imported and reconditioned and probably at least five years old. Several factors affect the supply and affordability of rural transport services in Africa^36^. Among them, Dennis et al.^36^ identify as key the fact that conventional vehicles designated to operate at high speeds on paved roads operate inefficiently at low speeds on rural earth roads where they are characterized by high fuel consumption and high emissions.

The study model calculates the emissions released during the transport of fuel to the consumption point as follows:

4 $${\text{E}}_{\text{v}} = {\text{E}}_{\text{fv}} \times \, {\text{c}} \, \times {\text{Tr}}_{\text{t}} \times \, 2{\text{t}}$$

where *E*_*v*_ represents the emissions released during the transport of fuel to the consumption point (in kilograms of carbon dioxide); *E*_*fv*_ is the emission factor for the vehicle (3.2 kg of carbon dioxide per litre^36^); *C* is the number of litres of diesel consumed by the van per hour (set to 14 l per hour^36^); *Tr*_*t*_ is the raster dataset in which the traveling time is expressed in seconds^35^; *2t* represents the times (both ways) that the van will travel to a particular pixel, which depends on the needed number of litres of diesel per year, calculated as follows:

5 $${\text{t}} \, = \, \left[ {\text{HH}}{_{\text{pix}} \times \, {\text{Dy}}_{\text{hh}} \times \, \eta \, } \right] \, / \, {\text{V}}$$

where η is the conversion efficiency; V the transported volume (using 0.286l per kWh and 300 l, respectively. Both parameters are derived from^4^).

Finally, the total emissions per pixel per year will result from adding *Ev* and *Ep*.

### Electricity consumption

In the simulation, a residential consumption level of 1,250 kWh per household per year is used with a low night consumption profile^23^ (Table S.5 includes household size per country). This number marks the threshold between Tier 3 and Tier 4 as defined by the Global Tracking Framework of the multitier matrix for measuring access to household electricity consumption^1^. This consumption level allows for general lighting, televisions, fans (as relevant), and any medium- to high-power appliances. In order to check the effect of different load profile, costs were calculated for a high night consumption profile (instead of a profile defined by two-thirds of the consumption occurring during daytime). The results of this sensitivity analysis are conclusive: costs increase by less than 10% in almost 80% of the area under study. In more than 90% of the locations, the difference in cost between low and high night-time consumption is less than 0.02 US$.

### Affordability among poor unelectrified households

To address the question of affordability, estimates are required, first, on the running costs of electricity and, second, on the amount of money that households can spend.

For the latter, the study relied on the Global Consumption Database^32^, which is a freely accessible database on household consumption patterns in developing countries. Started in 2007, the database is updated periodically and currently covers 92 developing countries. The data are drawn from national household surveys, which collect information for a group of households representative of the entire country. The available data refer to 2010, and, although one may not assume that consumption has not changed more recently, this is the only dataset with worldwide coverage on household consumption.

For each of the 92 countries covered, the market is segmented into four groups: the lowest consumption segment (the bottom half of the global distribution), the low-consumption segment (51th–75th percentiles), the middle consumption segment (76th–90th percentiles), and the highest consumption segment (to the 91st percentile and above). Some of the expenditure values in the Global Consumption Database are comparatively low. According to the data, the 4.5 billion low-income people in developing countries collectively spend more than US$5 trillion a year, more than the middle and higher consumption segments combined. The literature often states that households in developing countries spend a relatively high proportion of their disposable incomes on fuel^38,39^. In fact, according to the Global Consumption Database, the lower consumption segments spend significantly more than the middle and higher segments combined on energy and, especially, on food and beverages.

Because households actually spend their money on all types of fuels, it seems sensible to assume that they can afford to spend a share of their income on a particular fuel, as long as this fuel provides at least the same quality of service. This assumption ignores the substitution between energy and other goods purchased by households. Further exploration about the affordability definition is beyond the scope of this study. The present study focuses on the affordability of electricity use, rather than on the affordability of access (for example, the costs of connections), which would merit a separate effort. The affordability of use is usually approached by quantifying the share of household income allocated to energy^38^.

The analysis first examined Global Consumption Database data on household energy and electricity expenditures in the countries under study. Of the 71 countries, data for 49 countries are available. For the missing countries, the values reported on comparable countries are used on a case-by-base basis. Yearly expenditure data on electricity and energy (in millions of purchasing power parity U.S. dollars) for higher and lower segments and the population per country in each segment were obtained from the database. These data and data on household size were used to derive the yearly expenditure on electricity and energy per poor household, at the national level. These values show great variability. With US$11 spent on energy per year, the Comoros and Tanzania present the minimum values, while Pakistan, at US$420 per year, shows the maximum. In electricity, Togo and Liberia, at US$0.00 and US$0.23 per year are at the bottom, while Djibouti, at US$220 per year, shows the highest value. Understanding the variations not only between countries, but also within countries and the relation between increased energy spending and willingness to pay should be the object of dedicated studies. The literature on energy poverty in developing countries has not contributed sufficiently to knowledge on the factors associated with variations in energy spending^38^. The analysis that underlies this study has relied on the robustness of the methods used in the Global Consumption Database.

With regard to potential daily expenditures on electricity, the 80% of the current total expenditure on energy by the poor is used (for country values, refer to Table S.3). This represents the vast majority, if not the totality, of the unelectrified population in the countries under study. In sub-Saharan Africa in 2010, the same year as the year of the consumption data, the electricity access rate was 33.5%^20^. Therefore, electricity consumption does not provide an appropriate measure of the available purchasing power of households.

## Supplementary Information

Supplementary Information.
